# Brain volumes, behavioral inhibition, and anxiety disorders in children: results from the adolescent brain cognitive development study

**DOI:** 10.1186/s12888-024-05725-z

**Published:** 2024-04-04

**Authors:** Rawan A. Hammoud, Lara Abou Ammar, Stephen J. McCall, Wael Shamseddeen, Martine Elbejjani

**Affiliations:** 1 Faculty of Medicine, American University of Beirut, Beirut, Lebanon; 2 Department of Epidemiology and Population Health, Faculty of Health Sciences, American University of Beirut, Beirut, Lebanon; 3 Center for Research on Population and Health, Faculty of Health Sciences, American University of Beirut, Beirut, Lebanon; 4 Department of Psychiatry, Faculty of Medicine, American University of Beirut, Beirut, Lebanon; 5 Clinical Research Institute, Department of Internal Medicine, Faculty of Medicine, American University of Beirut, Beirut, Lebanon

**Keywords:** Behavioral inhibition, Brain volumes, Anxiety disorders, Children, Child development, Brain development

## Abstract

**Background:**

Magnetic resonance imaging (MRI) studies have identified brain changes associated with anxiety disorders (ADs), but the results remain mixed, particularly at a younger age. One key predictor of ADs is behavioral inhibition (BI), a childhood tendency for high avoidance of novel stimuli. This study aimed to evaluate the relationships between candidate brain regions, BI, and ADs among children using baseline data from the Adolescent Brain Cognitive Development (ABCD) study.

**Methods:**

We analyzed global and regional brain volumes of 9,353 children (9–10 years old) in relation to BI and current ADs, using linear mixed models accounting for family clustering and important demographic and socioeconomic covariates. We further investigated whether and how past anxiety was related to brain volumes.

**Results:**

Among included participants, 249 (2.66%) had a current AD. Larger total white matter (Beta = -0.152; 95% CI [-0.281, -0.023]), thalamus (Beta = -0.168; 95% CI [-0.291, -0.044]), and smaller hippocampus volumes (Beta = 0.094; 95% CI [-0.008, 0.196]) were associated with lower BI scores. Amygdala volume was not related to BI. Larger total cortical (OR = 0.751; 95% CI [0.580;0.970]), amygdala (OR = 0.798; 95%CI [0.666;0.956]), and precentral gyrus (OR = 0.802; 95% CI [0.661;0.973]) volumes were associated with lower odds of currently having ADs. Children with past ADs had smaller total white matter and amygdala volumes.

**Conclusions:**

The results show associations between brain volumes and both BI and ADs at an early age. Importantly, results suggest that ADs and BI have different neurobiological correlates and that earlier occurrences of ADs may influence brain structures related to BI and ADs, motivating research that can better delineate the similarities and divergence in the neurobiological underpinnings and building blocks of BI and ADs across their development in early life.

**Supplementary Information:**

The online version contains supplementary material available at 10.1186/s12888-024-05725-z.

## Background

Anxiety disorders (ADs) are the most prevalent class of psychiatric disorders across the lifespan worldwide [1]. They are recognized for their early onset and multiple comorbidities [2]. When occurring early and left untreated, ADs are associated with a higher risk of subsequent mood disorders, substance use disorder, suicidal ideation, educational underachievement, and economic disadvantage in adult years [3]. Moreover, untreated ADs exhibit a chronic course [4] and are associated with a low rate of spontaneous remission [5–7]. The significant burden associated with childhood-onset ADs underscores the importance of early prevention, detection, and management, all of which necessitate a more comprehensive understanding of the pathophysiology of the disorders’ onset and development. A key step in this direction is to identify earlier direct links between structural brain characteristics with behavioral predictors and clinical manifestations of ADs.

Advances in neuroimaging research have been identifying potential associations between structural changes in candidate brain regions and pediatric-onset ADs. However, to date, data have been extremely mixed and largely limited by smaller sample sizes (detailed review of published studies included in the Supplementary Material—Supplemental Table 1). Whereas some studies found evidence for larger amygdalar volumes [8, 9], others found smaller [10–12], or no difference in amygdalar volumes in youth with ADs [13, 14]. Similarly, while pediatric ADs were associated with decreased hippocampal volume in some studies [11, 13], there was no association in others [8, 14]. Some studies identified an increase in the putamen volume in children with ADs [15, 16], whereas others failed to identify any difference [8, 14]. The existing literature suggests that larger volumes of the insula [11], precuneus [17], precentral gyrus [17], and dorsal anterior cingulate [18] are related to ADs. Mixed results were detected in the prefrontal cortex [14, 17–20]. The lingering inconsistencies in results emphasize the need for further investigations in this area.

Behavioral inhibition (BI) is one of the most clearly described developmental risk factors for ADs [3, 21]. BI is an early-appearing component of temperament defined as a pattern of timidity and avoidance when faced with novel stimuli [21]. BI as conceptualized by Kagan and Gray focus on response to unfamiliar and unpredictable situations (which is distinct other common usage of the term referring to concepts of inhibitory control and withholding inappropriate response) [22]. Several neuroimaging studies have reported variations in certain brain structures, functions, and connectivity in individuals with BI. The results regarding brain structures have been largely heterogeneous (detailed review of published studies included in the Supplementary Material—Supplemental Table 2). Volumes of the amygdala [23, 24] and the hippocampus [23, 25, 26] were positively correlated with BI in some studies, but not in others [25–29]. Volumes of the caudate [24, 29] and the orbitofrontal cortex [27, 28] have been associated with BI scores, but with opposing directions across different studies. Other findings include an association between larger precuneus [27], putamen [29], and insula [29] volumes and lower BI and no association with the nucleus accumbens [28]. These mixed results can be explained by several factors, including a smaller sample size, different age groups investigated, and limited adjustment for important covariates, namely sociodemographic factors.

Previous research exploring the potential genetic etiology of anxiety disorders indicates a weak direct effect of common genetic variants on the development of childhood ADs [30]. Similarly, a recent analysis using data from the Adolescent Brain Cognitive Development (ABCD) study, the largest study in the U.S. assessing brain development, estimated a low heritability of BI in children [29]. However, the authors were able to demonstrate a relatively high heritability of some observed volumetric correlates of BI (ventral caudate, putamen, hypothalamus, right anterior insula, and a cluster in cerebellar vermis) [29]. Together, these findings motivate investigations that can delineate the neurobiological features contributing to the early development of both the behavioral risk factor and clinical AD.

This study aims to add to the understanding of the underlying pathophysiology of anxiety by using the large sample of the ABCD study, adjusting for socioeconomic indicators, and delineating important relationships between brain volumes, BI, and current and past ADs in children. We first examined the relationships of global and candidate brain regions with BI and current ADs among children; we then assessed whether and how past occurrences of ADs were related to the candidate brain regions.

## Methods

### Participants

This is a cross-sectional study using the first public release (2.0.1) of the Adolescent Brain Cognitive Development (ABCD) study (https://abcdstudy.org/index.html). The ABCD is an ongoing longitudinal study that was launched in September 2016 and that recruited over 11,000 children aged 9–10 years from 21 centers throughout the United States. Recruitment was designed to reflect as much as possible the sociodemographic diversity of the US population. Details of recruitment and study design were previously described [31]. For this study, the analytical sample consisted of 9,353 children with complete data on variables of interest (socio-demographic indicators, BI, childhood ADs) and with magnetic resonance imaging (MRI) images passing the quality control measures (Supplemental Fig. 1).

### Ethical considerations

ABCD research sites rely on a central Institutional Review Board (IRB) at the University of California, San Diego, for the ethical review and approval of the research protocol, with a few sites obtaining local IRB approval. Written and verbal consent was collected from both the parent/guardian and child before participating in the study [32]*.* The current study was also approved by the American University of Beirut IRB (SBS-2019–0467) for a secondary analysis of deidentified data.

### Measures

#### Brain volumes

Participants conducted a baseline MRI session using one of three 3T scanner platforms (General Electric 750, Siemens Prisma, or Philips Achieva and Ingenia lines) [33]. MRI acquisition parameters were previously described [34]. Scanner serial number was included as a random effect in analyses of past ADs where the outcome was brain volume. High-resolution T1-weighted structural MRIs were obtained. Images were processed by the ABCD study team using FreeSurfer version 5.3.0 (https://surfer.nmr.mgh.harvard.edu/fswiki/FreeSurferWiki), according to standard processing pipelines that addressed multiple challenges including head motion, distortion, and intensity in homogeneity [34]. Participants were excluded if they had poor quality T1 scans (iqc_t1_ok_ser = 0; *n* = 10), if FreeSurfer outputs did not pass a predetermined quality check (fsqc_qc = 0; *n* = 402), or if any incidental findings were noted from the neuroradiological read of the structural MRI images (mrif_score = 0 | mrif_score = 3 or mrif_score = 4; *n* = 416). This analysis used the z-scores of volumes (cm^3^) of whole brain (sum of gray and white matter), total cortical (GM), total cerebral white matter (WM) and sixteen predetermined regions of interest (amygdala, hippocampus, parahippocampus, insula, anterior cingulate, posterior cingulate, orbitofrontal cortex, prefrontal cortex, putamen, thalamus, cuneus, precuneus, nucleus accumbens, pallidum, entorhinal, and precentral gyrus; total sum of left and right volumes) based on their reported links to BI and/or ADs in the literature (Detailed review of published studies included in the Supplementary Material—Supplemental Tables 1 & 2). The mean volumes of the selected regions of interest are described in the Supplementary Material- Supplemental Table 3. We controlled for intracranial volume (ICV- sum of gray matter, white matter, meninges and cerebrospinal fluid) in statistical models as described below, consistent with previous ABCD studies. To note, we did not adjust for ICV in the main analysis of whole brain volumes and sensitivity analysis adjusting for ICV yielded similar conclusions.

#### Outcomes

##### *Behavioral inhibition*

BI was assessed using the behavioral inhibition/behavioral activation scale (BIS/BAS) developed by Carver and White (1994) [35, 36]. The BIS subscale includes seven items that reflect the sensitivity of an individual to possible aversive events. Responses are scored from 0 “not true” to 3 “totally true”, with the overall score for behavioral inhibition ranging from 0 (low level of inhibition) to 21 (high level of inhibition). The BIS/BAS scale was originally developed to measure personality traits in adults but was validated for use in children and adolescents [37–39]. Although the scale is a self-report, it accurately reflects tendencies to engage in approach/avoidance behavior [38, 40].

##### Childhood anxiety disorder

Children’s mental health was evaluated using the computerized version of the Kiddie Schedule for Affective Disorders and Schizophrenia for DSM-5 (KSADS-5) [36]. The children completed age-appropriate modules selected by the ABCD team based on expert opinions (mood disorders, separation anxiety, social anxiety, generalized anxiety, sleep, and suicidality), while caregivers completed the full interview. The KSADS is a reliable and valid measurement tool to measure psychopathology in children and adolescents [41, 42]. The abridged youth version may be used at ages as early as 9–10 years old with the support of trained staff, as was done in the ABCD study [36]. The computerized version has good reliability when compared to the clinician-administered version [43].

In childhood, generalized, separation, and social anxiety disorders are combined under one entity labeled the “pediatric anxiety triad” due to the shared risk factors, neurobiology, comorbidity, and response to treatment [3]. As such, in our study, a child met the criteria for current ADs if either the parent or the child reported that the child had any of the pediatric anxiety triad disorders. A history of past childhood anxiety disorder was similarly assessed by asking the parents to complete the KSADS questionnaire for past symptoms of anxiety in the child. The outcome for both current and past childhood ADs was a dichotomous outcome with 0 for absence and 1 for the presence of disorder.

##### *Covariates*

Both biological, including sex, and socioeconomic factors, including household income and parental education, have been individually associated with childhood BI [44, 45], ADs [44, 46], and brain development [47–50]. The ABCD study collected sociodemographic data from the children’s parents. Our study included adjustments for the following covariates, given their links to BI, AD, and brain development in prior research and in the ABCD sample (Supplementary Tables 5– 7): age, sex assigned at birth, race, ethnicity, parental marital status, family income, and parents' education [34].

Parents’ psychopathology (any and anxiety-specific) was adjusted for due to evidence that it may influence the presence and their report on the child’s mental health [51]. It was assessed using the Adult Self Report from the Achenbach System of Empirically Based Assessment (ASEBA) for adults. A T-score of 65 was considered to be in the clinical range [52]. A dichotomous variable was created with 0 for the absence and 1 for the presence of psychopathology.

##### *Statistical analysis*

Sample characteristics were reported using the mean and standard deviation (SD) for continuous variables and frequency (%) for categorical variables. First, we estimated bivariate analyses between the covariates of interest and each of BI, ADs, and volume of the cortical gray matter using linear mixed models, while accounting for clustering within the family unit since 28.87% of our analytical sample had at least one sibling participating in the study. The association between each of the global and region of interest volumes (exposure) and BI (outcome) was examined through a linear mixed model with accounting for clustering within families. Three models were assessed with stepwise adjustment for ICV, age, sex, race, parent education, parent relationship, household income (model 1), current childhood anxiety disorders (model 2), and past childhood anxiety disorders (model 3). The association between brain volumes (exposure) and current ADs (outcome) was examined using multilevel mixed-effects logistic regression and accounting for clustering within families. This analysis included four models: first we adjusted for age, sex, race, parent education, parent relationship, household income, and ICV (model 1), then we additionally adjusted for past anxiety (model 2), current parent anxiety disorder (model 3), and BI scores (model 4). A sensitivity analysis (model 5) adjusted for the presence of any current parental psychopathology (Supplementary Material – Supplemental Table 8). Additional analysis was performed to estimate the relationship between past ADs (exposure) and the brain volumes of interest (outcome), using linear mixed models with family and MRI device serial numbers as random effects and adjusted for age, sex, race, parent education, parent relationship status, household income, and ICV. We have also conducted additional analyses investigating any differential associations with left and right global and regional brain volumes (Supplementary Material – Supplemental Tables 9, 10, 11). *P*-values less than 0.05 were considered statistically significant. All statistical analyses were performed using STATA version 13.1 (STATA Corp LP, College Station, Texas, USA).

## Results

The mean age was 119 months (SD = 7.47); 52.12% of the sample were males, 19.22% were Hispanic, 13.23% were non-Hispanic black, and 55.30% of children were non-Hispanic white. The majority of children lived with married parents (69.92%); 62% of the children had at least one parent with a college degree or above and 42.48% had a combined family annual income above $100,000 (Table 1). In our final sample, 2.66% of children had current ADs and 14.08% had a past ADs diagnosis (Table 1). BI score was normally distributed with an average of 9.51 (SD = 3.73). Compared with the excluded sample (participants with missing data on variables of interest), the analytical sample had a higher proportion of non-Hispanic White participants, family income of more than $50,000, and married parents with at least a college degree. Children with an anxiety disorder had significantly higher BI scores (Supplemental Fig. 2). Flow chart descriptive of the sample and brain volumes as well as bivariate analysis between BI and AD and between sociodemographic covariates and each of BI, current ADs, and gray matter volume are presented in the Supplementary Material- Supplemental Tables 4– 7.

**Table 1:** Descriptive statistics of the study sample (*N* = 9 353)

Demographics	N	Mean ± SD
Age	9 353	119.00 (7.47)
	N	Valid %
Sex	9 353	
Males	4 875	52.12%
Females	4 478	47.88%
Race	9 353	
Non-Hispanic White	5 172	55.30%
Non-Hispanic Black	1 237	13.23%
Non-Hispanic Asian	176	1.88%
Non-Hispanic others/mixed	970	10.37%
Hispanic	1 798	19.22%
Income	9 353	
< 50 K	2 677	28.62%
50-100 K	2 703	28.90%
100 K +	3 973	42.48%
Parent highest education	9 353	
Less than a college degree	3 516	37.59%
College degree and above	5 837	62.41%
Parent marital status	9 353	
Married	6 540	69.92%
Widowed	76	0.81%
Divorced/Separated	1 181	12.63%
Never married	1 060	11.33%
Living with partner	496	5.30%
Parent anxiety	9 353	5.67%
Parent any psychopathology	9 353	19.60%
Outcomes		
Behavioral inhibition score	9 353	9.51 (3.73)
Child Anxiety		
Present	249	2.66%
Past	1 317	14.08%

### Brain volumes and behavioral inhibition

Larger thalamic volume (beta = -0.168; 95% CI [-0.291; -0.044]) and cerebral WM volume (beta = -0.152; 95% CI [-0.281; -0.023]) were associated with lower BI (Table 2). Results remained similar following adjustment for current and past child ADs. Larger putamen volume showed a pattern of association with lower BI scores (beta = -0.089; 95% CI [-0.183;0.005]) while a larger hippocampal volume had a pattern of association with higher BI scores (beta = 0.094; 95% CI [-0.008;0.196]) across tested models.

**Table 2:** Separate models for multivariable analysis of brain volumes (z-scores) and behavioral inhibition scores, adjusting for sociodemographic indicators and child’s anxiety disorder

*Candidate brain structure*	Model 1		Model 2		Model 3	
	**β**	**95% CI**	**β**	**95% CI**	**β**	**95% CI**
Whole brain	0.042	-0.051 0.135	0.048	-0.044 0.141	0.049	-0.044 0.142
Total cortical	0.003	-0.141 0.146	0.012	-0.131 0.156	0.009	-0.135 0.152
Total cerebral WM	**-0.152**	**-0.281 -0.023**	**-0.148**	**-0.277 -0.019**	**-0.142**	**-0.270 -0.013**
Total amygdala	0.038	-0.061 0.138	0.045	-0.054 0.144	0.046	-0.052 0.145
Total hippocampus	0.094	-0.008 0.196	0.095	-0.007 0.197	0.099	-0.002 0.202
Total accumbens	-0.005	-0.089 0.079	-0.0005	-0.084 0.083	0.001	-0.083 0.085
Total insula	-0.071	-0.176 0.035	-0.065	-0.171 0.039	-0.066	-0.171 0.039
Total anterior cingulate	-0.039	-0.123 0.044	-0.037	-0.120 0.046	-0.038	-0.121 0.045
Total posterior cingulate	-0.040	-0.136 0.056	-0.036	-0.132 0.060	-0.033	-0.129 0.063
Total orbitofrontal	-0.012	-0.111 0.087	-0.009	-0.108 0.090	-0.011	-0.110 0.088
Total prefrontal cortex	-0.001	-0.121 0.119	0.004	-0.116 0.125	0.004	-0.116 0.124
Total caudate	-0.056	-0.146 0.035	-0.052	-0.143 0.038	-0.055	-0.146 0.035
Total putamen	-0.089	-0.183 0.005	-0.091	-0.185 0.003	-0.087	-0.181 0.007
Total thalamus	**-0.168**	**-0.291 -0.044**	**-0.172**	**-0.295 -0.049**	**-0.163**	**-0.286 -0.039**
Total precuneus	0.076	-0.030 0.182	0.080	-0.026 0.186	0.079	-0.027 0.185
Total cuneus	-0.045	-0.136 0.046	-0.042	-0.133 0.049	-0.045	-0.136 0.046
Total entorhinal	0.011	-0.072 0.095	0.013	-0.071 0.096	0.011	-0.073 0.094
Total Parahippocampal	0.044	-0.044 0.131	0.047	-0.041 0.134	0.043	-0.044 0.131
Total precentral	-0.068	-0.175 0.038	-0.061	-0.168 0.045	-0.066	-0.172 0.041
Total pallidum	-0.019	-0.111 0.072	-0.017	-0.108 0.075	-0.020	-0.111 0.071

Model 1- age, sex, ICV, race, parent education, income, relationship status

Model 2- age, sex, ICV, race, parent education, income, relationship status, current anxiety disorder

Model 3- age, sex, ICV, race, parent education, income, relationship status, past anxiety disorder

Whole brain volume models do not include adjustment for ICV; sensitivity analyses including adjustment for ICV yielded similar conclusions

In bold: associations with a *p*-value < 0.05. Underlined: *p*-value close to significance 0.05 < *p*-value ≤ 0.1

*CI* confidence interval

### Brain volumes and current childhood anxiety disorders

Larger volumes of the total cortex (OR = 0.751; 95% CI [0.580;0.970]), amygdala (OR = 0.798; 95% CI [0.666;0.956]), and precentral gyrus (OR = 0.802; 95% CI [0.661;0.973]) were associated with lower odds of having a current ADs (Table 3). These associations remained significant after adjusting for present parent anxiety disorder (model 3) and behavioral inhibition (model 4). The observed results were similar in a sensitivity analysis adjusting for any parental psychopathology (Supplementary material- Supplemental Table 8). Adjusting for child past ADs (Model 2) diluted the statistical significance of the observed association for total cortical volume and amygdala but the magnitude and direction of the association remained. The volumes of the nucleus accumbens (OR = 0.872; 95% CI [0.749;1.016) and the insula (OR = 0.846; 95% CI [0.699;1.024]) showed a trend for an inverse association with anxiety (model 1) before adjusting for past child or present parent anxiety.

**Table 3:** Separate models for multivariable analysis of brain volumes (z-scores) and currently having an anxiety disorder, adjusting for sociodemographic indicators, behavioral inhibition score, child’s history of anxiety disorder, and presence of parental anxiety disorder

***Candidate brain structure***	Model 1		Model 2		Model 3		Model 4	
	**OR**	**95% CI**	**OR**	**95% CI**	**OR**	**95% CI**	***OR***	**95% CI**
Whole brain	0.825	0.698 0.975	0.856	0.719 1.020	0.818	0.691 0.969	0.821	0.694 0.971
Total cortical	**0.751**	**0.580 0.970**	0.766	0.584 1.004	**0.749**	**0.577 0.973**	**0.755**	**0.582 0.975**
Total cerebral WM	0.882	0.700 1.112	0.949	0.745 1.208	0.869	0.688 1.098	0.898	0.713 1.131
Total amygdala	**0.798**	**0.666 0.956**	0.841	0.695 1.019	**0.789**	**0.657 0.949**	**0.791**	**0.659 0.949**
Total hippocampus	0.956	0.797 1.146	0.995	0.823 1.202	0.948	0.789 1.138	0.945	0.787 1.133
Total accumbens	0.872	0.749 1.016	0.899	0.765 1.058	0.879	0.753 1.026	0.871	1.066 1.144
Total insula	0.846	0.699 1.024	0.862	0.710 1.047	0.851	0.702 1.032	0.854	0.706 1.033
Total anterior cingulate	0.937	0.807 1.089	0.946	0.806 1.111	0.927	0.796 1.079	0.945	0.813 1.098
Total posterior cingulate	0.875	0.734 1.043	0.906	0.752 1.091	0.878	0.734 1.049	0.883	0.741 1.052
Total orbitofrontal	0.913	0.765 1.089	0.911	0.758 1.096	0.912	0.764 1.089	0.915	0.766 1.091
Total prefrontal cortex	0.849	0.685 1.054	0.870	0.694 1.092	0.854	0.687 1.061	0.853	0.687 1.058
Total caudate	0.902	0.768 1.059	0.881	0.745 1.041	0.900	0.765 1.059	0.910	0.775 1.069
Total putamen	1.060	0.897 1.253	1.082	0.910 1.286	1.046	0.882 1.239	1.075	0.909 1.271
Total thalamus	1.115	0.896 1.386	1.181	0.937 1.488	1.104	0.885 1.378	1.134	0.912 1.410
Total precuneus	0.887	0.733 1.072	0.907	0.743 1.107	0.902	0.744 1.094	0.883	0.730 1.069
Total cuneus	0.919	0.781 1.079	0.902	0.759 1.073	0.920	0.781 1.084	0.924	0.786 1.086
Total entorhinal	0.962	0.828 1.118	0.959	0.819 1.124	0.966	0.829 1.125	0.960	0.826 1.117
Total Parahippocampal	0.906	0.769 1.067	0.902	0.759 1.073	0.899	0.762 1.061	0.900	0.764 1.061
Total precentral	**0.802**	**0.661 0.973**	**0.813**	**0.665 0.994**	**0.793**	**0.652 0.965**	**0.811**	**0.668 0.984**
Total Pallidum	0.913	0.775 1.076	0.909	0.767 1.078	0.899	0.761 1.062	0.917	0.777 1.082

Model 1- age, sex, ICV, race, parent education, income, relationship status

Model 2- age, sex, ICV, race, parent education, income, relationship status, past child anxiety disorder

Model 3- age, sex, ICV, race, parent education, income, relationship status, parent anxiety disorder

Model 4- age, sex, ICV, race, parent education, income, relationship status, behavioral inhibition scores

Whole brain volume models do not include adjustment for ICV; sensitivity analyses including adjustment for ICV yielded similar conclusions

In bold: associations with a *p*-value < 0.05. Underlined: *p*-value close to significance 0.05 < *p*-value ≤ 0.1

*CI* confidence interval

### Past anxiety and brain volumes

In the analysis of how past occurrences of anxiety relate to brain volumes, the results showed that past ADs were associated with smaller whole brain (beta = -6.907; 95% CI [-12.156; -1.658]), cerebral WM (beta = -1.737; 95% CI [-2.961;-0.512]), and amygdala volumes (beta = -0.019; 95% CI [-0.036;-0.001]) (Table 4).

**Table 4:** Multivariable analysis of past anxiety disorder (predictor) and candidate brain volumes (outcome, in cm3), adjusting for sociodemographic indicators

***Candidate brain structure***	Past Anxiety	
	**β**	**95% CI**
Whole-brain	**-6.907**	**-12.156 -1.658**
Cortical Gray	-0.993	-2.442 0.457
White matter	**-1.737**	**-2.961 -0.512**
Amygdala	**-0.019**	**-0.036 -0.001**
Hippocampus	-0.029	-0.064 0.006
Nucleus accumbens	-0.004	-0.013 0.005
Insula	-0.060	-0.125 0.005
Anterior cingulate	-0.021	-0.125 0.082
Postetrior cingulate	-0.044	-0.097 0.009
Orbitofrontal cortex	-0.009	-0.128 0.108
Prefrontal	-0.446	-1.016 0.124
Caudate	0.001	-0.046 0.049
Putamen	-0.011	-0.069 0.048
Thalamus	-0.031	-0.081 0.018
Precuneus	-0.029	-0.154 0.096
Cuneus	-0.008	-0.061 0.046
Entorhinal	0.004	-0.030 0.038
Parahippocampus	0.004	-0.033 0.041
Precentral gyrus	-0.043	-0.185 0.099
Pallidum	0.005	-0.013 0.023

Whole brain volume models do not include adjustment for ICV; sensitivity analyses including adjustment for ICV yielded similar conclusions

In bold: associations with a *p*-value < 0.05. Underlined: *p*-value close to significance 0.05 < *p*-value ≤ 0.1

*CI* confidence interval

Additional analyses showed that all reported associations are observed with both left and right volumes of global and regional brain volumes, indicating a consistent pattern of symmetry. We note that one negative association was observed between the left precentral volume (and not right precentral volume) and BI scores (Supplementary Material – Supplemental Tables 9, 10 and 11).

## Discussion

**Fig. 1:**
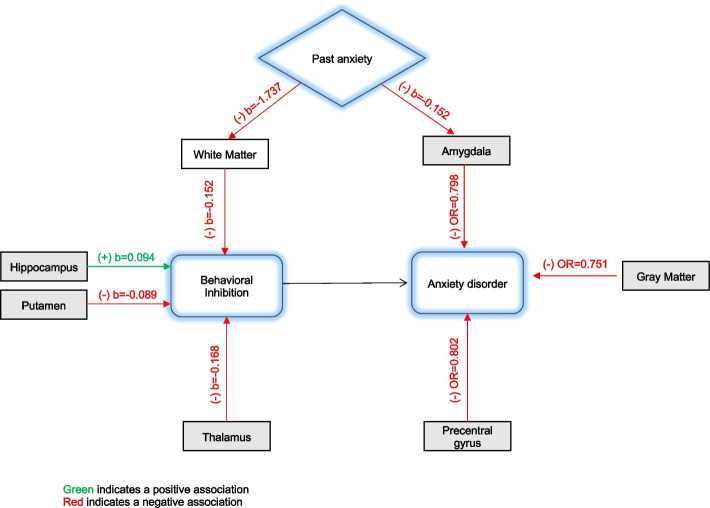
Summary of findings

While substantial progress has been achieved in understanding the neurobiological and temperament risk factors for anxiety disorders, the interplay between these pathways remains largely unclear. We used data from the ABCD study, the largest study of brain development and child health in the United States, to evaluate structural brain variations related to ADs and the temperamental risk factor, BI, in middle childhood age. We found that larger cerebral WM and thalamic volumes were associated with lower BI scores. Different brain regions, notably larger cortical gray matter, amygdala, and precentral gyrus volumes were associated with a lower risk of ADs. These results suggest divergence in neurostructural variations linked to BI and ADs. Our findings also showed that past anxiety was associated with smaller WM and amygdala volumes suggesting that previous occurrences of ADs may influence brain development in a way that can increase the risk for BI and future ADs (Fig. 1).

### The hippocampus and the amygdala

In the functional neuroscience literature, it has been demonstrated that the hippocampus and the amygdala play an important role in the modulation of negative emotion and anxiety-related behaviors [53–55]. However, the results regarding the role of their volumetric properties are mixed. Consistent with some earlier studies, our results did not show an association between BI and the volume of the amygdala [25–29]. Two studies to date reported a positive relationship between amygdala volume and BI [23, 24]. These two studies had a relatively small sample size (*n* = 84 and *n* = 63 respectively), with the latter being restricted to only male participants. Participants in both studies were in their young adult years. In addition, the scales used to measure BI were different than those used in our study (sensitivity to punishment, and self-report of inhibition scales). Although different scales show strong correlations with each other [56], they may represent different underlying pathophysiology.

Regarding the hippocampus, most of the previous studies, except for two [27, 29], reported a positive correlation between hippocampal volume and BI [23, 25, 26]. In our sample, this relation approached significance after correction for the sociodemographic confounders. Ide et al. reported no association between BI and the hippocampus in the ABCD study [29]; their associations were only adjusted for age, ICV, and scanner model. BI has low levels of heritability during childhood suggesting a role for environmental factors in contributing to the manifestation and/or stability of BI across development [28]. Evidence exists on the influence of gender, socioeconomic status, parent psychopathology, and early social interactions on a child’s behavior [21, 42, 55, 56]. Aligned with these findings, we found that all demographic, socioeconomic, and family-related factors were associated with BI (Supplementary Material- Supplemental Table 5), with important indications that children of parents with higher socioeconomic status (higher income and educational level) had lower BI scores and lower odds for AD. Children of married parents and parents without anxiety disorders had significantly lower BI scores. All these factors (except for parental anxiety disorder) were also related to higher gray matter. These findings emphasize the importance of accounting for parental/family socioeconomic indicators in future studies of brain volumes, BI, and ADs and of prioritizing their role in research, intervention, and prevention efforts.

The odds of having current ADs were inversely associated with the volume of the amygdala but not with the hippocampus. Our results regarding the amygdala are in line with previous animal [57] and human pediatric [10–12, 18] and adult studies [58, 59]. Similar to our results, previous studies did not report associations between ADs and hippocampal volume [8, 10, 14]. Two pediatric samples found a smaller hippocampal volume in children with ADs [11, 13]. These variations can be explained by a mixture of small sample sizes, slightly older age of participants, and inclusion of participants with any anxiety disorder rather than being limited to the pediatric triad as was done in this study. Given that adolescence is an age of transition when most brain structures undergo drastic modifications [60], even a couple of years in difference can impact the associations observed. Hence, it is important to study the neuroanatomical basis of anxiety at different ages.

BI and ADs were differently associated with the hippocampus and the amygdala. The hippocampus has been suggested to have a role in risk assessment aspects of anxiety [61]. The amygdala on the other hand had a greater role in increased arousal and active avoidance [61, 62]. As such, the hippocampus and the amygdala may potentially contribute differently to anxiety. Consequently, this may explain how different underlying neural mechanisms result in BI, a risk factor for anxiety, and clinical ADs.

### The thalamus

To the best of our knowledge, this is the first study to report an association between larger thalamic volume and lower BI. The thalamus plays an integral role in information transfer and assimilation in the human brain [63]. It is an essential hub in the transfer of sensory information to the limbic system, particularly the amygdala [64]. It also has a role in social-emotional development, threat attention mediation [65], and regulation of chronic stress and anxiety-like behavior in animal and human studies [66–68]. Consistent with our findings, a smaller thalamic volume has been reported in adult patients with social anxiety [69] and panic disorders [70]. Although the amygdala and the hippocampus have been studied more extensively in relation to inhibited temperament due to their role in fear processing, the brain remains a complicated system of interconnected pathways. This observed association with the thalamus, a region that extensively communicates with the amygdala, in this younger age may represent an earlier timestamp in the associations between the amygdala and anxiety and/or an underlying link between ADs and BI.

### Other brain regions

Other brain regions associated with BI in this study were the white matter and the putamen. A smaller putamen volume was similarly related to BI in the study performed by Ide et al. using the ABCD sample [29]. Prior sMRI studies (Supplementary Material- Supplemental Table 2) did not analyze associations between BI and WM [23, 27]. However, in line with our findings, a recent diffusion tensor imaging study concluded that children with higher global WM microstructure (an indicator of effective neural communication [71] had lower levels of general psychopathology [71].

Regarding anxiety, the cortical gray matter and precentral volumes were significantly associated with present anxiety. None of the previous studies on childhood anxiety either investigated or reported associations with these regions. Results regarding cortical gray matter suggest that beyond links to a few brain structures, the association of ADs and the brain may be widespread, motivating future replications and investigations on this finding and its potential ramifications. The precentral gyrus primarily has a motor function. Some studies have suggested that activity in this region may influence emotional processing [72]. Some fMRI studies also found increased activity in the precentral gyrus in individuals with depression and anxiety disorders [73, 74].

Overall, these findings show that both BI and ADs have neuroanatomical correlates at a younger age with no overlap between these correlates, suggesting potentially divergent neurobiological pathways underlying BI and ADs rather than a common neurobiological pathway leading to both temperamental and clinical risk. Other possibilities may be that common neurobiological risk trajectories for temperament and ADs are not yet strongly established in childhood or that they are more complex and converge at different points, as suggested by the results regarding prior experiences of ADs.

### Past anxiety and brain volumes

Previous occurrences of ADs were associated with smaller whole brain, WM, and amygdala volumes. This again indicated a potential widespread link between ADs and brain volumes. At the same time, given that WM and amygdala were related to current BI and ADs, respectively, this suggests that previous anxiety may shape a trajectory of risk for BI and future ADs through these neurobiological links. BI has been suggested to have a discontinuous pattern from infancy to adolescence [75]. Multiple factors are hypothesized to shape BI across the years, including cognitive processes, parenting styles, and the caregiving environment [75]. Our results further suggest that previous occurrences of ADs may have a role in reinforcing a neurobiological underpinning and a continuous pattern for behavioral inhibition and the development of an anxiety disorder. The associations observed at this early age between structural brain changes, BI, and past and current anxiety motivate longitudinal studies on the stepwise build-up of their connections. It also prompts future studies to identify modifiable factors contributing to these connections and to the continuity of BI and ADs.

### Clinical implications

The development of ADs is a complex process involving behavioral, neurobiological, and environmental processes. These factors have mostly been studied separately, making it difficult to parse out their relationships and convergence. Findings from this study support the presence of earlier neurobiological-behavioral links and suggest that they might have direct ramifications on behavioral and clinical AD risk. This highlights the importance of regular screening for behavioral changes in children and of taking into consideration previous occurrences of ADs for earlier detection of higher and more continual risk for ADs. A special focus on teaching children with risk factors (higher BI and/or previous AD) and their parents healthy coping mechanisms may aid in preventing the development or recurrence of the disorder. As previous ADs are linked to both higher behavior- and brain-related increased risk for subsequent ADs, it is important to identify optimal and longer-term approaches to break this loop and prevent recurrence of ADs.

Second, studies from the adult population report changes in the neural architecture in response to psychotherapeutic interventions [76]. Understanding the principal brain regions at play in the development of ADs might guide future psychotherapeutic trials in monitoring responses to interventions.

Third, this study brings to attention the important role of some potentially modifiable socioeconomic and family-related factors (namely family income and parental education and relationship status) for the development of both the behavioral risk factor and ADs, highlighting the value of comprehensive approaches incorporating social and parental support and awareness.

### Strengths and limitations

The study’s strengths include the large sample size and adjustment for important sociodemographic and family/parent indicators. In addition, the use of the ABCD Study, a nationally representative sample, increases the generalizability of the findings. Moreover, instead of lifetime diagnoses, our study examined both past and current anxiety disorders, based on the participant and/or their caregiver’s response to the KSAD questionnaire of symptoms. The study also had several limitations. This was a cross-sectional study in which inference about developmental trajectories or causality cannot be made. Nonetheless, the results obtained inform about the presence and absence of associations between specific brain volumes and BI and anxiety disorders and strengthen the rationale for potential studies using future ABCD data releases to investigate the temporal sequence of the associations observed. The analytical sample had a significantly higher proportion of children coming from families with higher income and parental educational level, which may limit the inference of the findings to other socioeconomic groups. The associations observed in our study persisted even after adjusting for socioeconomic and sociodemographic factors. However, we note that factors such as higher income and parental educational level were related to lower BI, lower risk of AD, and higher gray matter volumes in bivariate analysis (Supplementary Tables 5– 7); associations observed in our sample may thus be modulated with more diverse sepctra of BI and gray matter volumes. Additionally, we did not have information about the age of onset for the current ADs or the duration of previous anxiety. Given our finding that a history of ADs is associated with brain volumes in this age group, understanding and accounting for the role of ADs duration is essential in future studies. Although the BIS/BAS has been reported to accurately reflect tendencies to engage in approach/avoidance behavior [38, 40], it remains a self-reported instrument with a risk of recall bias. Finally, the covariates we controlled for are all essential to BI, ADs, and brain development; however, they are complex constructs that are difficult to measure, and residual confounding could be present.

## Conclusion

In a large sample of children, total brain WM, thalamus, putamen, and hippocampus were associated with behavioral inhibition, with the thalamus being a novel region described in this study. Variations in different brain regions, notably the gray matter, precentral gyrus, and amygdala were associated with the risk of ADs at this young age. These findings suggest that different neurobiological features may be related to anxiety and its temperamental risk factor, BI, motivating future work on the similarities and divergence of neurological underpinnings and building blocks of BI and ADs. Our results also highlight the role of prior occurrences of anxiety disorders in influencing the brain and regions that were related to BI and ADs. Longitudinal studies are needed to examine how BI, anxiety, and brain volumes intersect at various stages of early life and development to better mitigate the formation of longer-term and/or adverse feedback loops jeopardizing both brain and mental health.

### Supplementary Information

**Supplementary Material 1.**

## Data Availability

Data used in the preparation of this article were obtained from the Adolescent Brain Cognitive Development^SM^ (ABCD) Study (https://abcdstudy.org), held in the NIMH Data Archive (NDA).

## Abbreviations

ABCD: Adolescent Brain Cognitive Development
AD: Anxiety Disorder
ASEBA: Adult Self Report from the Achenbach System of Empirically Based Assessment
BI: Behavioral Inhibition
BIS/BAS: Behavioral Inhibition/Behavioral Activation Scale
GM: Gray Matter
ICV: Intracranial Volume
IRB: Institutional Review Board
KSADS-5: Kiddie Schedule for Affective Disorders and Schizophrenia for DSM-5
MRI: Magnetic resonance imaging
SD: Standard Deviation
WM: White Matter
