# Supplementary material for: Brain volumes, behavioral inhibition, and anxiety disorders in children: results from the adolescent brain cognitive development study
Source: BMC Psychiatry. 2024 Apr 4;24:257. doi: 10.1186/s12888-024-05725-z (PMC10996182; doi:10.1186/s12888-024-05725-z)
Supplement: Supplementary file 1 — Supplementary Material 1. [file 12888_2024_5725_MOESM1_ESM.docx]

Supplementary material

Content

**Supplemental Table 1.** Summary of the literature on the associations between brain volumes and pediatric anxiety.

**Supplemental Table 2.** Summary of the literature on the association between brain volumes and behavioral inhibition

**Supplemental Table 3.** Descriptive analysis of included regions of interest

**Supplemental Table 4.** Descriptive analysis of total and excluded participants

**Supplemental Table 5.** Unadjusted bivariate analysis with behavioral inhibition as outcome

**Supplemental Table 6.** Bivariate analysis with cortical gray matter as outcome, adjusted for intracranial volume

**Supplemental Table 7.** Unadjusted bivariate analysis with child's current anxiety as outcome

**Supplemental Table 8.** Sensitivity analysis for the association of current child anxiety with brain volumes (in cm3), adjusting for age, sex, intracranial volume, race, parent education, income, relation status, presence of any parent psychopathology

**Supplemental Table 9.** Multivariable analysis of BI with left and right brain volumes (z-scores in cm3), adjusting for sociodemographic parameters and child’s anxiety

**Supplemental Table 10.** Multivariable analysis of current child anxiety with left and right brain volumes (in cm3), adjusting for sociodemographic parameters, BIS, child and parent’s anxiety

**Supplemental Table 11.** Multivariable analysis of candidate brain left and right volumes(outcome) (in cm3) with past anxiety (predictor), adjusting for sociodemographic parameters

**Supplemental Figure 1.** Flowchart for exclusion criteria.

**Supplemental Figure 2.** Box plot of BI score by presence or absence of anxiety

**Supplemental Table 1.** Summary of the literature on the associations between brain volumes and pediatric anxiety.

AD: anxiety disorders, H: healthy, GAD: generalized anxiety disorder,
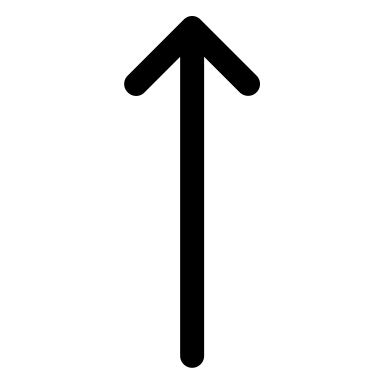
:larger,
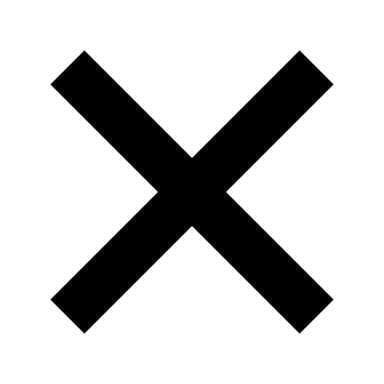
: no difference,
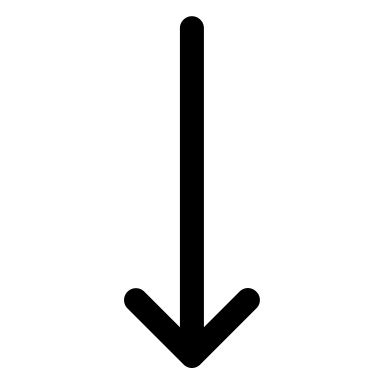
:smaller

| Study | Sample size | Disorder | Mean age (y) | Brain volumes |
| --- | --- | --- | --- | --- |
| De Bellis et al. (2000) ^8^ | 36 *(12 AD, 24 H)* | GAD | 12 | 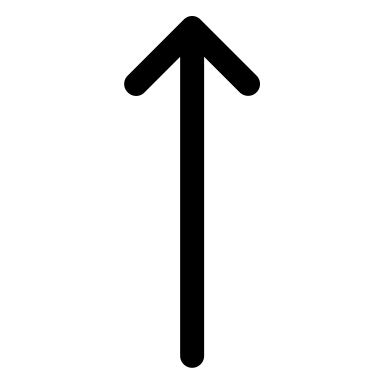: Amygdala  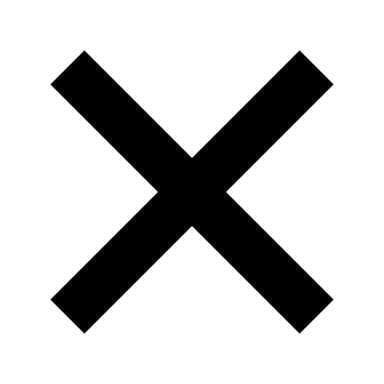: Intracranial, cerebral gray and white matter, temporal lobe, hippocampal, caudate, putamen |
| De Bellis et al. (2002) ^20^ | 111 *(13 AD, 98 H)* | GAD | 12.5 | 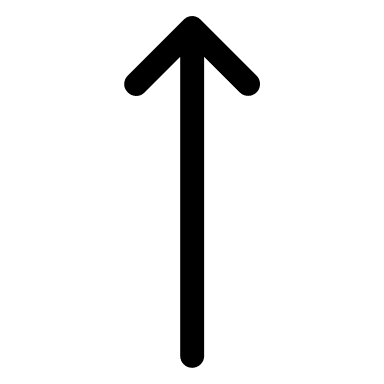: Superior temporal gyrus  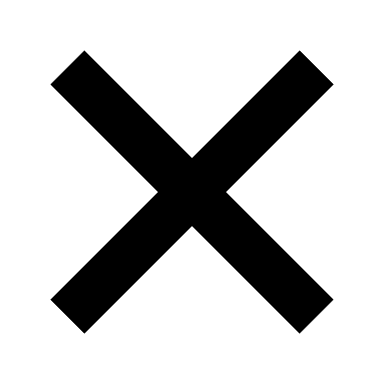: Thalamus and prefrontal lobe |
| Milham et al. (2005)^10^ | 51 *(17 AD, 34 H)* | Mixed AD | 13 | 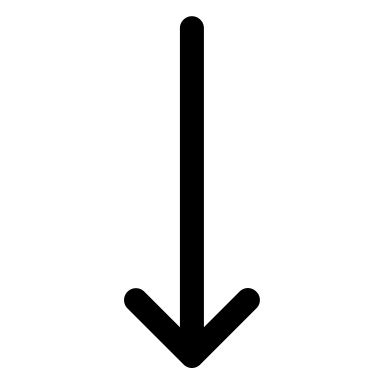: Left amygdala  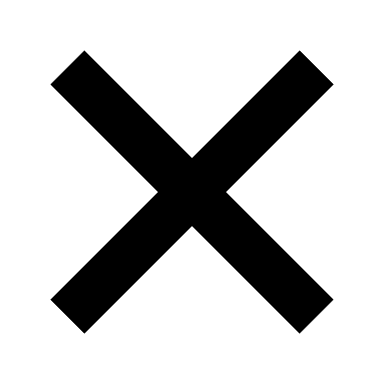: All other brain regions |
| Strawn et al. (2013) ^17^ | 28 *(15 AD, 13 H)* | GAD | 13 | 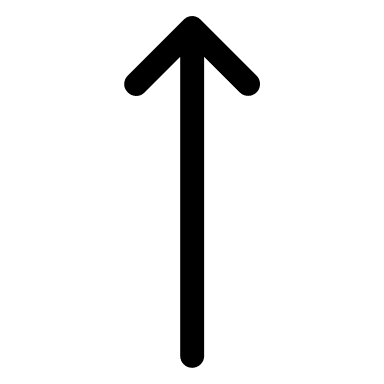: Right precuneus; right precentral gyrus, white matter of the left inferior temporal gyrus.  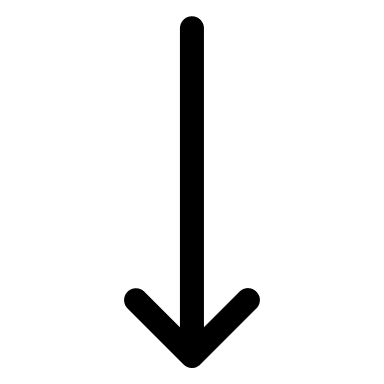: Left orbital gyrus and posterior cingulate, white matter of the left medial and superior frontal gyrus |
| Mueller et al. (2013)^11^ | 102 *(39 AD, 63 H)* | Mixed AD | 13 | 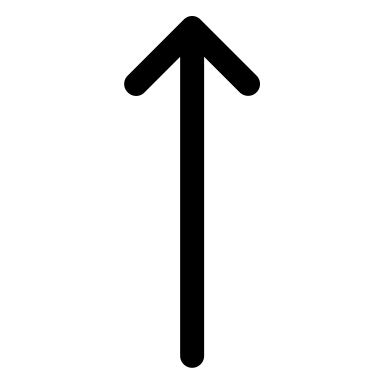: Insula  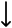: Amygdala and anterior hippocampal volumes. |
| Liao et al. (2013)^15^ | 51 *(26 AD, 25 H)* | GAD | 17 | 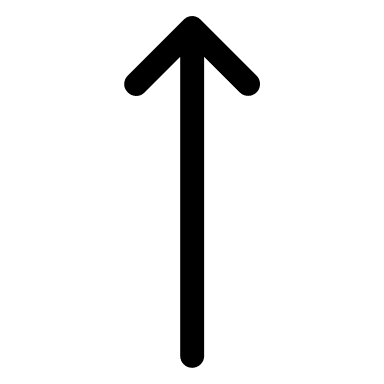: Right putamen |
| Strawn et al. (2014) ^12^ | 32 *(13 AD, 19 H)* | Mixed AD | 14 | 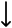: Amygdala |
| Liao et al. (2014) ^16^ | 51 *(26 AD, 25 H)* | GAD | 17 | 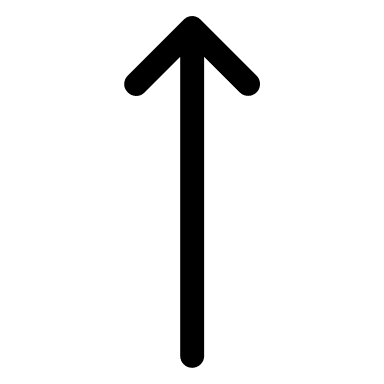: Right putamen |
| Qin et al. (2014) ^9^ | 76 | Mixed AD | 8.2 | 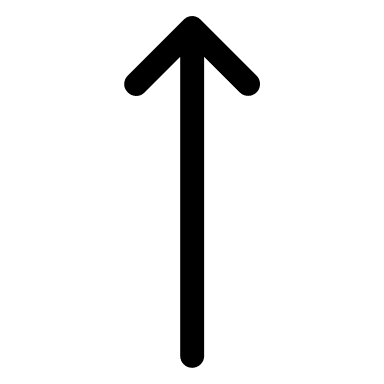: Left amygdala  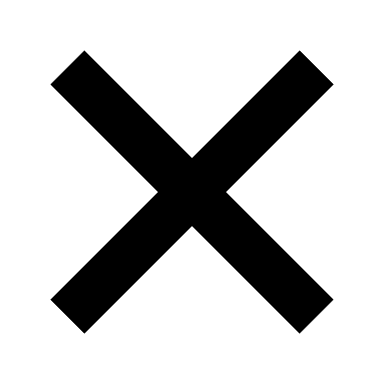: Total gray and white matter. |
| Strawn et al. (2015) ^18^ | 65 *(38 AD, 27 H)* | Mixed AD | 14 | 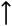: Dorsal anterior cingulate  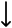: Inferior frontal gyrus (ventrolateral prefrontal cortex), postcentral gyrus, cuneus/precuneus, and amygdala. |
| Gold et al. (2016) ^19^ | 184 *(39 AD, 53 H, 92 other psychiatric diseases)* | Mixed AD | 13 | 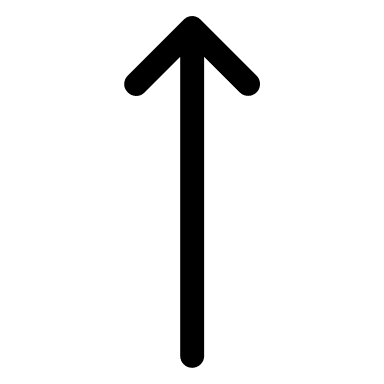: left dorsolateral prefrontal cortex |
| Gold et al. (2017) ^13^ | 151 *(75 AD, 76 H)* | Mixed AD | 12 | 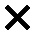: Amygdala  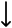: Right hippocampal |
| Merz et al. (2018)^14^ | 327 | Mixed AD | 13.65 | 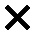: any of the subcortical regions of interest (amygdala, hippocampus, caudate, putamen, nucleus accumbens, pallidum, prefrontal cortex). |

**Supplemental Table 2.** Summary of the literature on the association between brain volumes and behavioral inhibition.

BI: Behavioral inhibition, H: healthy control,
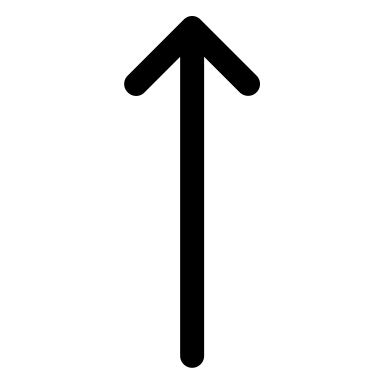
:larger,
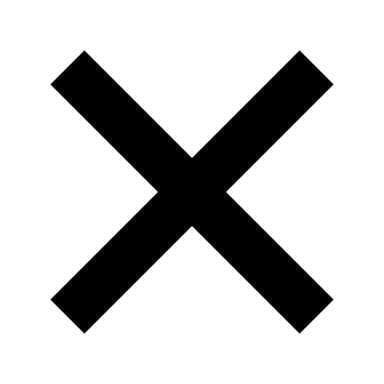
: no difference,
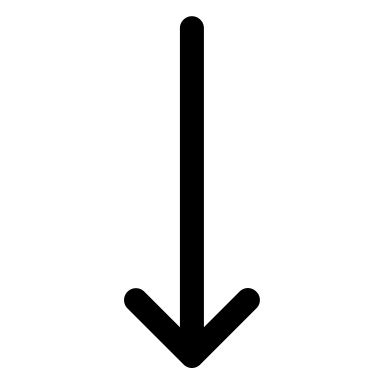
:smaller

| **Study** | **N** | **Age range (y)** | **BI scale** | **Covariates** | **Brain volumes** |
| --- | --- | --- | --- | --- | --- |
| **Barrós-Loscertales et al. (2006) ^22^** | 63 *(only males)* | 18-34 | Sensitivity to Punishment scale | Total brain volume | 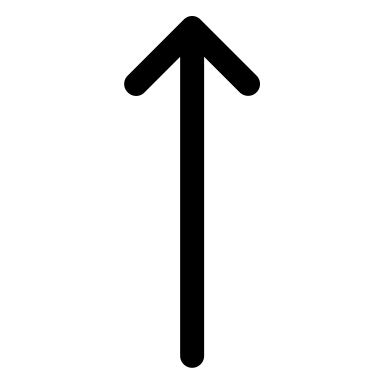: Bilateral parahippocampus, right amygdala, and hippocampus. |
| **Cherbuin et al. (2008)** **^24^** | 430 | 44-48 | Carver and White BIS/BAS scale | Age, sex, education, intracranial volume, and total brain volume | 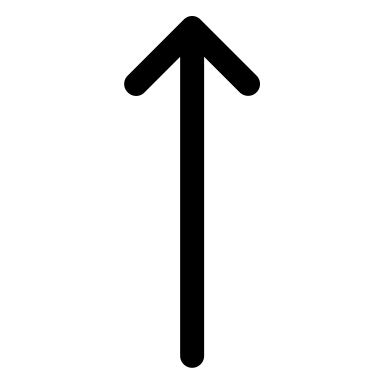: Hippocampus  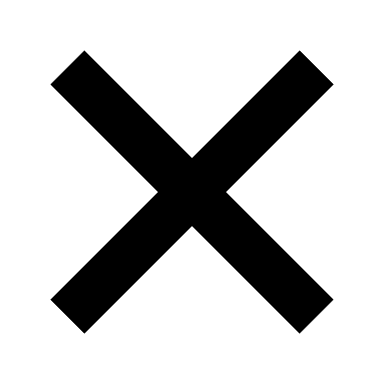: Amygdala |
| **Fuentes et al. (2012) ^26^** | 114 *(only males)* | 18-53 | Carver and White BIS/BAS scale | Age | 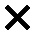: amygdala and hippocampus  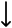: Precuneus and orbitofrontal cortex |
| **Urošević et al. (2012) ^27^** | 184 | 9.21-23.96 | Carver and White BIS/BAS scale | Age, sex, MRI scanner upgrade status | 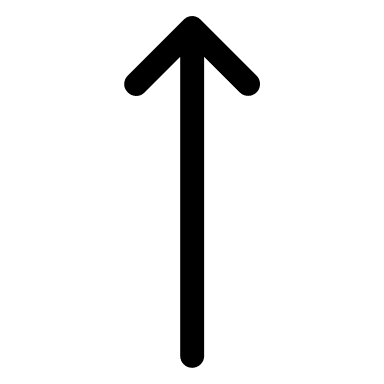: Left lateral orbitofrontal  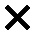: Medial orbitofrontal cortex, nucleus accumbens, amygdala |
| **Clauss et al. (2014) ^23^** | 84 *(40 BI, 44 H)* | Mean=23.4 | Retrospective Self-Report of Inhibition (RSRI) and the Adult Self-Report of Inhibition (ASRI) | Age, sex, race, handedness, intracranial volume | 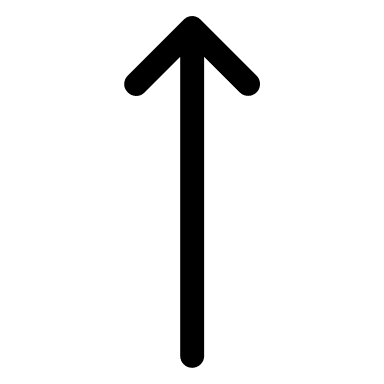: amygdala and left caudate |
| **Levita et al. (2014) ^25^** | 30 | 18-32 | Sensitivity to Punishment scale | Age, sex, intracranial volume | 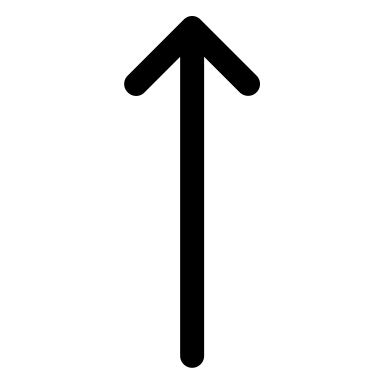: Right hippocampus  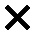: Amygdala |
| **Ide et al. (2020) ^28^** | 11,542 *(sample from ABCD study)* | 9-10 | Pagliaccio modified BIS/BAS scale | Age, intracranial volume, MRI scanner model | 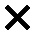: Orbitofrontal and amygdala  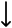: Ventral caudate, putamen, hypothalamus, right anterior insula, cluster in cerebellar vermis *(after accounting for BAS)* |

**Supplemental Table 3.** Descriptive analysis of included regions of interest.

| Volumes | N | Mean±SD (cm^3^) |
| --- | --- | --- |
| Whole-brain (Total GM+WM) | 9,259 | 1211.00 (113.09) |
| Intracranial (whole brain, CSF, meninges) | 9,353 | 1517.06 (148.97) |
| Total GM cortical volume | 9,353 | 596.79 (56.83) |
| Total Cerebral WM | 9,353 | 418.27 (48.87) |
| Total Amygdala | 9,353 | 3.19 (0.43) |
| Total Hippocampus | 9,353 | 8.15 (0.82) |
| Total accumbens | 9,353 | 1.19 (0.19) |
| Total Insula | 9,353 | 15.22 (1.69) |
| Total anterior cingulate | 9,353 | 10.75 (2.01) |
| Total posterior cingulate | 9,353 | 8.14 (1.19) |
| Total Orbitofrontal | 9,353 | 30.66 (3.19) |
| Total Prefrontal cortex | 9,353 | 189.06 (18.43) |
| Total Precuneus | 9,353 | 25.88 (3.19) |
| Total Caudate | 9,353 | 8.19 (1.04) |
| Total Putamen | 9,353 | 11.76 (1.29) |
| Total Thalamus | 9,353 | 14.99 (1.39) |
| Total Cuneus | 9,353 | 7.32 (1.16) |
| Total entorhinal | 9,353 | 3.71 (0.67) |
| Total Parahippocampal | 9,353 | 5.20 (0.75) |
| Total precentral | 9,353 | 32.13 (3.56) |

**Supplemental Table 4.** Descriptive analysis of total and excluded participants

| Demographics | Excluded (2,522) | Analytical sample (9,353) |  | Total sample (11,875) |
| --- | --- | --- | --- | --- |
|  | Mean±SD | Mean±SD | p-value | Mean±SD |
| Age | 118.73 (7.42) | 119.00 (7.47) | 0.103 | 118.94 (7.46) |
|  | Valid % | Valid % |  |  |
| Sex |  |  |  |  |
| Males | 52.19% | 52.12% | 0.955 | 52.14% |
| Females | 47.81% | 47.88% |  | 47.86% |
| Race |  |  |  |  |
| Non-Hispanic white | 40.11% | 55.30% | <0.001 | 52.08% |
| Non-Hispanic Black | 21.60% | 13.23% |  | 15.00% |
| Non-Hispanic Asian | 3.02% | 1.88% |  | 2.12% |
| Non-Hispanic others/mixed | 11.00% | 10.37% |  | 10.50% |
| Hispanic | 24.27% | 19.22% |  | 20.29% |
| Income |  |  |  |  |
| <50K | 36.24% | 28.62% | <0.001 | 29.68% |
| 50-100K | 24.40% | 28.90% |  | 28.28% |
| 100K+ | 39.36% | 42.48% |  | 42.05% |
| Parent highest education |  |  |  |  |
| Less than a college degree | 51.30% | 37.59% | <0.001 | 40.49% |
| College degree and above | 48.70% | 62.41% |  | 59.51% |
| Parent marital status |  |  |  |  |
| Married | 59.82% | 69.92% | <0.001 | 67.84% |
| Widowed | 0.87% | 0.81% |  | 0.82% |
| Divorced/Separated | 15.02% | 12.63% |  | 13.12% |
| Never married | 16.34% | 11.33% |  | 12.36% |
| Living with partner | 7.96% | 5.30% |  | 5.85% |

**Supplemental Table 5.** Unadjusted bivariate analysis with behavioral inhibition as outcome

CI, confidence interval

| Independent variables | beta | p-value | 95% CI |
| --- | --- | --- | --- |
| Age | 0.003 | 0.556 | -00.7 0.013 |
| Sex (female) | **0.651** | <0.001 | 0.498 0.803 |
| Race (reference: Non-Hispanic White) |  |  |  |
| Non-Hispanic Black | 0.231 | 0.056 | -0.006 0.468 |
| Non-Hispanic Asian | 0.444 | 0.125 | -0.123 1.011 |
| Non-Hispanic others/mixed | 0.224 | 0.094 | -0.038 0.486 |
| Hispanic | **0.367** | <0.001 | 0.163 0.571 |
| Income (reference: <50k) |  |  |  |
| 50-100k | -0.046 | 0.658 | -0.250 0.158 |
| 100k+ | **-0.305** | 0.001 | -0.493 -0.118 |
| Parent education: College degree and above | **-0.262** | 0.001 | -0.422 -0.102 |
| Parent marital status (reference: Married) |  |  |  |
| Widowed | -0.802 | 0.071 | -1.674 0.069 |
| Divorced/Separated | 0.229 | 0.058 | -0.008 0.465 |
| Never married | 0.185 | 0.143 | -0.062 0.433 |
| Living with partner | **0.475** | 0.007 | 0.127 0.822 |
| Current anxiety | **1.338** | <0.001 | 0.870 1.807 |
| Past anxiety | **0.622** | <0.001 | 0.445 0.880 |
| Parent anxiety | **0.679** | <0.001 | 0.346 1.014 |

**Supplemental Table 6.** Bivariate analysis with cortical gray matter as outcome, adjusted for intracranial volume

CI, confidence interval

| Independent variables | beta | p-value | 95% CI |
| --- | --- | --- | --- |
| Age | **-0.705** | <0.001 | -0.774 -0.636 |
| Sex (female) | **-1.936** | 0.002 | -0.316 -0.712 |
| Race (reference: Non-Hispanic White) |  |  |  |
| Non-Hispanic Black | **-21.054** | <0.001 | -22.871 -19.237 |
| Non-Hispanic Asian | **-9.949** | <0.001 | -13.991 -5.907 |
| Non-Hispanic others/mixed | **-9.878** | <0.001 | -11.748 -8.008 |
| Hispanic | **-9.559** | <0.001 | -11.237 -7.881 |
| Income (reference: <50K) |  |  |  |
| 50-100 | **6.639** | <0.001 | 5.129 8.150 |
| 100+ | **9.831** | <0.001 | 8.374 11.287 |
| Parent education: College degree and above | **6.903** | <0.001 | 5.686 8.119 |
| Parent marital status (reference: Married) |  |  |  |
| Widowed | -6.195 | 0.052 | -12.448 0.058 |
| Divorced/Separated | **-3.337** | <0.001 | -5.047 -1.628 |
| Never married | **-11.133** | <0.001 | -12.975 -9.292 |
| Living with partner | **-7.105** | <0.001 | -9.615 -4.595 |
| Current anxiety | -3.027 | 0.068 | -6.279 0.224 |
| Past anxiety | -0.494 | 0.526 | -2.018 1.031 |
| Bis | -0.099 | 0.166 | -0.241 0.041 |
| Parent Anxiety | -1.051 | 0.392 | -3.459 1.357 |

**Supplemental Table 7.** Unadjusted bivariate analysis with child's current anxiety as outcome

CI, confidence interval

| Independent variables | OR | p-value | 95% CI |
| --- | --- | --- | --- |
| Age | 0.988 | 0.186 | 0.970 1.006 |
| Sex (female) | 1.086 | 0.541 | 0.832 1.418 |
| Race (reference: Non-Hispanic White) |  |  |  |
| Non-Hispanic Black | 1.273 | 0.214 | 0.869 1.864 |
| Non-Hispanic Asian | 0.206 | 0.122 | 0.028 1.522 |
| Non-Hispanic others/mixed | 1.074 | 0.751 | 0.688 1.677 |
| Hispanic | 0.947 | 0.771 | 0.660 1.361 |
| Income (reference: <50K) |  |  |  |
| 50-100K | 0.763 | 0.123 | 0.542 1.075 |
| 100K+ | **0.712** | 0.035 | 0.519 0.977 |
| Parent education: College degree and above | **0.596** | <0.001 | 0.456 0.780 |
| Parent marital status (reference: Married) |  |  |  |
| Widowed | 0.484 | 0.493 | 0.061 3.852 |
| Divorced/Separated | 1.399 | 0.085 | 0.955 2.049 |
| Never married | 1.474 | 0.052 | 0.997 2.182 |
| Living with partner | 1.083 | 0.797 | 0.590 1.988 |
| BIS | **1.106** | <0.001 | 1.068 1.145 |
| Past anxiety | **18.947** | <0.001 | 13.358 26.874 |
| Parent Anxiety | **5.474** | <0.001 | 3.714 8.070 |

**Supplemental Table 8.** Sensitivity analysis for the association of current child anxiety with brain volumes (in cm3), adjusting for age, sex, intracranial volume, race, parent education, income, relation status, presence of any parent psychopathology

CI, confidence interval

| **Candidate brain structure** | **OR** | **P** | **95% CI** |
| --- | --- | --- | --- |
| Whole brain | 0.855 | 0.068 | 0.722 1.012 |
| Total cortical | **0.760** | **0.040** | **0.586 0.987** |
| Total cerebral WM | 0.887 | 0.315 | 0.701 1.121 |
| Total amygdala | **0.805** | **0.022** | **0.669 0.969** |
| Total hippocampus | 0.971 | 0.756 | 0.809 1.166 |
| Total accumbens | 0.888 | 0.133 | 0.760 1.037 |
| Total insula | 0.848 | 0.095 | 0.699 1.029 |
| Total anterior cingulate | 0.936 | 0.390 | 0.804 1.089 |
| Total posterior cingulate | 0.876 | 0.147 | 0.733 1.047 |
| Total orbitofrontal | 0.917 | 0.342 | 0.768 1.096 |
| Total prefrontal cortex | 0.856 | 0.164 | 0.689 1.065 |
| Total caudate | 0.900 | 0.205 | 0.765 1.059 |
| Total putamen | 1.056 | 0.524 | 0.892 1.251 |
| Total thalamus | 1.124 | 0.298 | 0.902 1.401 |
| Total precuneus | 0.919 | 0.393 | 0.759 1.115 |
| Total cuneus | 0.910 | 0.264 | 0.772 1.073 |
| Total entorhinal | 0.968 | 0.676 | 0.830 1.128 |
| Total Parahippocampal | 0.897 | 0.200 | 0.759 1.059 |
| Total precentral | **0.803** | **0.028** | **0.659 0.977** |
| Total pallidum | 0.919 | 0.327 | 0.778 1.087 |

*Whole brain volume models do not include adjustment for ICV; sensitivity analyses including adjustment for ICV yielded similar conclusions.*

*In bold: associations with a p-value <0.05. Underlined: p-value close to significance 0.05<p-value≤0.1*

**Supplemental Table 9.** Multivariable analysis of BI with left and right brain volumes (z-scores in cm3), adjusting for sociodemographic parameters and child’s anxiety

| ***Candidate brain structure*** | **Model 1** | |
| --- | --- | --- |
|  | **β** | **95% CI** |
| Whole brain |  |  |
| Left cortical | -0.006 | -0.151 0.138 |
| Right cortical | 0.012 | -0.130 0.153 |
| Left cerebral WM | **-0.149** | **-0.278 -0.021** |
| Right cerebral WM | **-0.152** | **-0.281 -0.023** |
| Left amygdala | 0.036 | -0.060 0.132 |
| Right amygdala | 0.029 | -0.064 0.122 |
| Left hippocampus | 0.091 | -0.006 0.188 |
| Right hippocampus | 0.074 | -0.026 0.174 |
| Left accumbens | 0.014 | -0.066 0.095 |
| Right accumbens | -0.022 | -0.107 0.063 |
| Left insula | -0.041 | -0.142 0.058 |
| Right insula | -0.084 | -0.188 0.019 |
| Left anterior cingulate | -0.031 | -0.122 0.060 |
| Right anterior cingulate | -0.039 | -0.123 0.044 |
| Left posterior cingulate | -0.017 | -0.107 0.074 |
| Right posterior cingulate | -0.039 | -0.127 0.047 |
| Left orbitofrontal | -0.013 | -0.112 0.085 |
| Right orbitofrontal | -0.008 | -0.102 0.085 |
| Left prefrontal cortex | 0.006 | -0.114 0.126 |
| Right prefrontal cortex | -0.005 | -0.121 0.112 |
| Left caudate | -0.056 | -0.146 0.035 |
| Right caudate | -0.052 | -0.141 0.037 |
| Left putamen | -0.084 | -0.177 0.008 |
| Right putamen | -0.082 | -0.175 0.011 |
| Left thalamus | **-0.143** | **-0.267 -0.019** |
| Right thalamus | **-0.152** | **-0.264 -0.041** |
| Left precuneus | 0.033 | -0.067 0.134 |
| Right precuneus | 0.095 | -0.006 0.196 |
| Left cuneus | -0.044 | -0.130 0.041 |
| Right cuneus | -0.029 | -0.117 0.059 |
| Left entorhinal | 0.014 | -0.067 0.095 |
| Right entorhinal | 0.004 | -0.077 0.085 |
| Left Parahippocampal | 0.056 | -0.025 0.138 |
| Right parahippocampal | 0.009 | -0.077 0.096 |
| Left precentral | **-0.117** | **-0.217 -0.016** |
| Right precentral | 0.004 | -0.094 0.103 |
| Left pallidum | -0.002 | -0.089 0.085 |
| Right pallidum | -0.031 | -0.117 0.055 |

*CI, Confidence interval*

*In bold: associations with a p-value <0.05. Underlined: p-value close to significance 0.05<p-value≤0.1*

**Supplemental Table 10.** Multivariable analysis of current child anxiety with left and right brain volumes (in cm3), adjusting for sociodemographic parameters, BIS, child and parent’s anxiety

| ***Candidate brain structure*** | Model 1 | |
| --- | --- | --- |
|  | **OR** | **95% CI** |
| Whole brain |  |  |
| Left cortical | **0.766** | **0.592 0.992** |
| Right cortical | **0.740** | **0.574 0.954** |
| Left cerebral WM | 0.888 | 0.705 1.118 |
| Right cerebral WM | 0.877 | 0.697 1.106 |
| Left amygdala | **0.784** | **0.659 0.932** |
| Right amygdala | 0.870 | 0.735 1.029 |
| Left hippocampus | 0.957 | 0.807 1.136 |
| Right hippocampus | 0.966 | 0.808 1.153 |
| Left accumbens | 0.920 | 0.796 1.064 |
| Right accumbens | **0.848** | **0.727 0.989** |
| Left insula | 0.863 | 0.719 1.035 |
| Right insula | 0.859 | 0.712 1.035 |
| Left anterior cingulate | 0.965 | 0.820 1.136 |
| Right anterior cingulate | 0.937 | 0.807 1.089 |
| Left posterior cingulate | 0.828 | 0.699 0.980 |
| Right posterior cingulate | 0.979 | 0.837 1.145 |
| Left orbitofrontal | 0.922 | 0.773 1.099 |
| Right orbitofrontal | 0.920 | 0.778 1.087 |
| Left prefrontal cortex | 0.836 | 0.673 1.037 |
| Right prefrontal cortex | 0.883 | 0.717 1.087 |
| Left caudate | 0.914 | 0.778 1.074 |
| Right caudate | 0.897 | 0.765 1.051 |
| Left putamen | 1.096 | 0.929 1.293 |
| Right putamen | 1.014 | 0.861 1.196 |
| Left thalamus | 1.101 | 0.884 1.372 |
| Right thalamus | 1.099 | 0.903 1.340 |
| Left precuneus | 0.945 | 0.789 1.131 |
| Right precuneus | 0.864 | 0.722 1.035 |
| Left cuneus | 0.915 | 0.785 1.066 |
| Right cuneus | 0.949 | 0.812 1.109 |
| Left entorhinal | 0.969 | 0.839 1.121 |
| Right entorhinal | 0.970 | 0.837 1.124 |
| Left Parahippocampal | 0.959 | 0.825 1.117 |
| Right parahippocampal | 0.878 | 0.739 1.044 |
| Left precentral | 0.884 | 0.735 1.062 |
| Right precentral | **0.787** | **0.656 0.944** |
| Left pallidum | 0.900 | 0.767 1.057 |
| Right pallidum | 0.968 | 0.831 1.128 |

*CI, Confidence interval*

*In bold: associations with a p-value <0.05. Underlined: p-value close to significance 0.05<p-value≤0.1*

**Supplemental Table 11.** Multivariable analysis of candidate brain left and right volumes(outcome) (in cm3) with past anxiety (predictor), adjusting for sociodemographic parameters

| ***Candidate brain structure*** | Past Anxiety | |
| --- | --- | --- |
|  | **β** | **95% CI** |
| Whole brain |  |  |
| Left cortical | -0.464 | -1.196 0.268 |
| Right cortical | -0.525 | -1.256 0.205 |
| Left cerebral WM | **-0.856** | **-1.469 -0.241** |
| Right cerebral WM | **-0.879** | **-1.497 -0.261** |
| Left amygdala | -0.009 | -0.019 0.001 |
| Right amygdala | -0.009 | -0.019 0.001 |
| Left hippocampus | -0.009 | -0.029 0.009 |
| Right hippocampus | -0.019 | -0.038 0.001 |
| Left accumbens | -0.002 | -0.007 0.003 |
| Right accumbens | -0.002 | -0.007 0.003 |
| Left insula | -0.032 | -0.067 0.003 |
| Right insula | -0.027 | -0.063 0.009 |
| Left anterior cingulate | -0.018 | -0.069 0.033 |
| Right anterior cingulate | -0.011 | -0.062 0.041 |
| Left posterior cingulate | -0.034 | -0.066 0.001 |
| Right posterior cingulate | -0.011 | -0.046 0.024 |
| Left orbitofrontal | -0.006 | -0.071 0.059 |
| Right orbitofrontal | -0.002 | -0.068 0.064 |
| Left prefrontal cortex | -0.197 | -0.498 0.103 |
| Right prefrontal cortex | -0.249 | -0.553 0.054 |
| Left caudate | 0.006 | -0.018 0.031 |
| Right caudate | -0.005 | -0.029 0.020 |
| Left putamen | -0.001 | -0.034 0.032 |
| Right putamen | -0.009 | -0.037 0.019 |
| Left thalamus | -0.019 | -0.046 0.007 |
| Right thalamus | -0.012 | -0.039 0.014 |
| Left precuneus | -0.012 | -0.082 0.057 |
| Right precuneus | -0.016 | -0.088 0.055 |
| Left cuneus | 0.003 | -0.027 0.033 |
| Right cuneus | -0.008 | -0.042 0.025 |
| Left entorhinal | 0.001 | -0.019 0.022 |
| Right entorhinal | 0.003 | -0.019 0.024 |
| Left Parahippocampal | -0.009 | -0.032 0.015 |
| Right parahippocampal | 0.013 | -0.008 0.035 |
| Left precentral | -0.015 | -0.099 0.068 |
| Right precentral | -0.026 | -0.108 0.056 |
| Left pallidum | -0.001 | -0.014 0.012 |
| Right pallidum | 0.006 | -0.003 0.015 |

*CI, Confidence interval*

*In bold: associations with a p-value <0.05. Underlined: p-value close to significance 0.05<p-value≤0.1*

**Supplemental Figure 1.** Flowchart for exclusion criteria.

BIS: Behavioral inhibition system; KSAD: Kiddie Schedule for Affective Disorders and Schizophrenia; ASR: Adult Self Report.


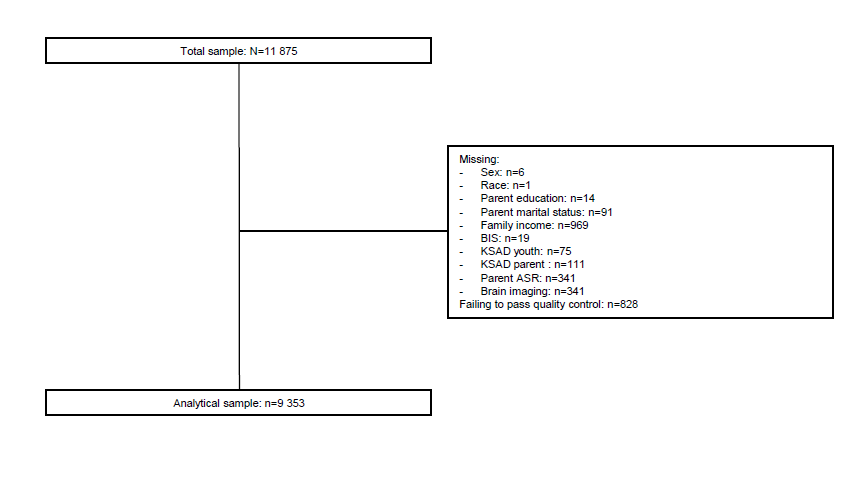


**Supplemental Figure 2.** Box plot of BI score by presence or absence of anxiety


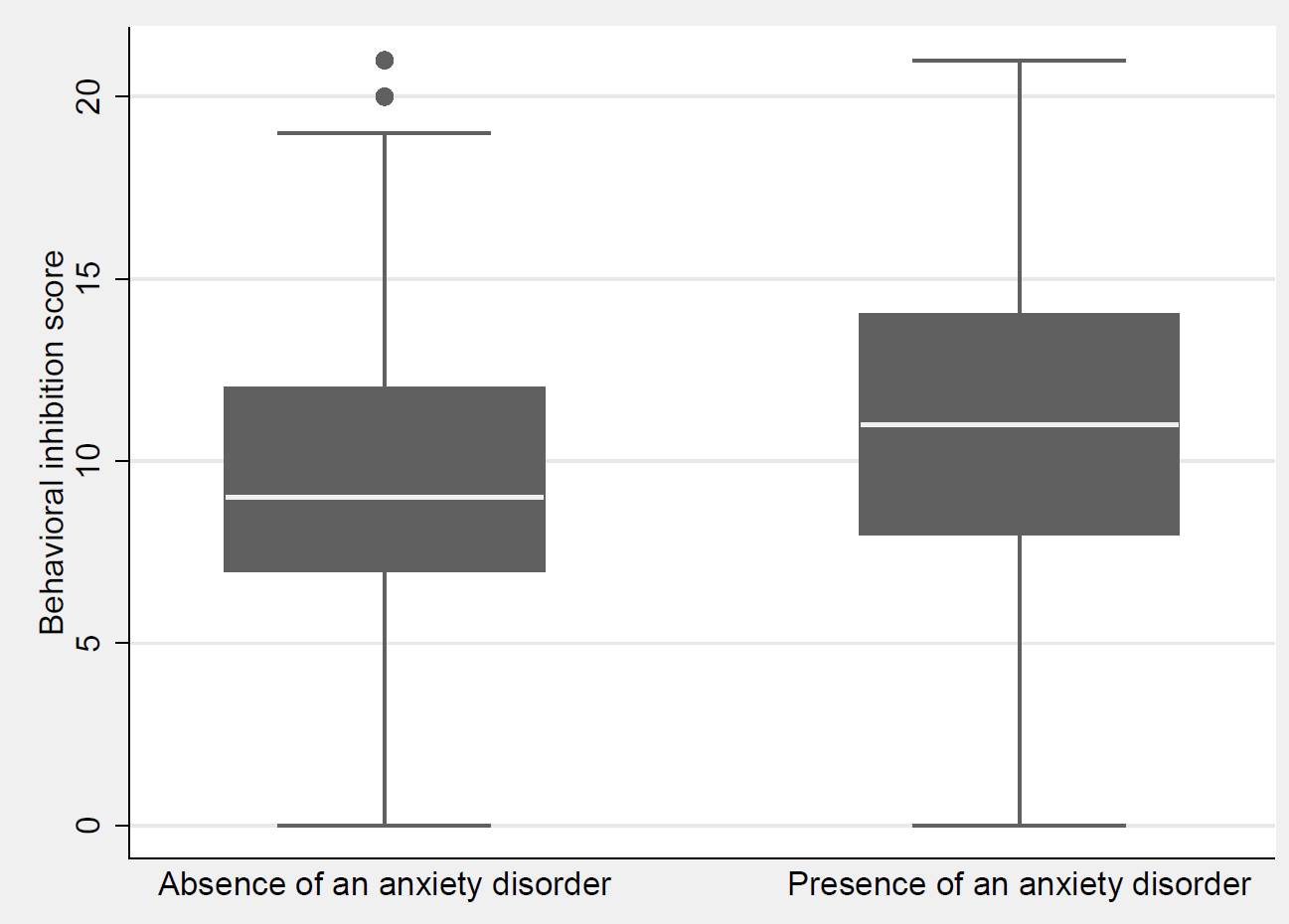


*Legend: Behavioral inhibition assessed using the behavioral inhibition/behavioral activation scale (BIS/BAS) developed by Carver and White (1994). Responses are scored from 0 “not true” to 3 “totally true”, with the overall score for behavioral inhibition ranging from 0 (low level of inhibition) to 21 (high level of inhibition).*
